# Supplementary material for: Substrate-Specific Activation of α-Secretase by 7-Deoxy-Trans-Dihydronarciclasine Increases Non-Amyloidogenic Processing of β-Amyloid Protein Precursor
Source: Molecules. 2020 Feb 3;25(3):646. doi: 10.3390/molecules25030646 (PMC7037359; doi:10.3390/molecules25030646)
Supplement: Supplementary file 1 [file molecules-25-00646-s001.pdf]

Substrate-specific activation of α-secretase by 7-deoxy-trans-dihydronarciclasine increases non-amyloidogenic processing of amyloid-β protein precursor

Yoon Sun Chun[1, 2#], Yoon Young Cho[2#], Oh Hoon Kwon[2], Dong Zhao[1], Hyun Ok Yang[1, 3*,] Sungkwon Chung[2*]

[1]Natural Products Research Center, Korea Institute of Science and Technology, Gangneung, Gangwon-do, South Korea
[2]Department of Physiology, Samsung Biomedical Research Institute, Sungkyunkwan University School of Medicine, Suwon, South Korea
[3]Department of Biological Chemistry, University of Science & Technology (UST), Daejeon, South Korea

Supplementary Fig. 1

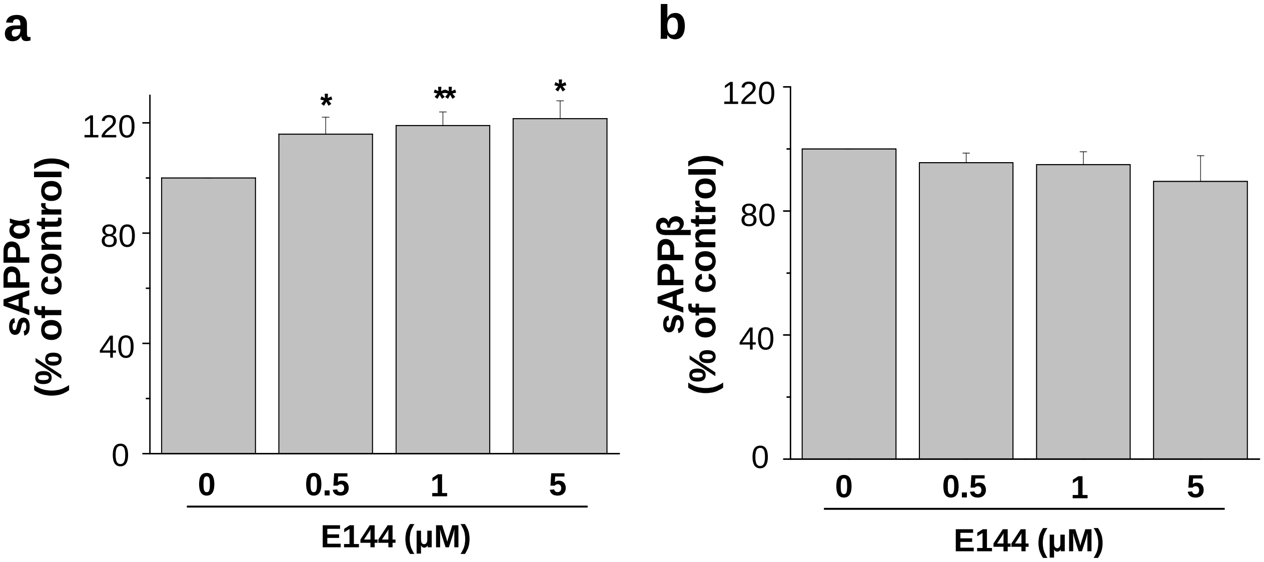

Supplementary Figure 1. E144 increased the secretion of sAPPα and decreased Aβ from SH-SY5Y cells stably transfected with wild type APP. Cells were incubated with E144 for 1 h. Levels of sAPPα (a) or sAPPβ (b) in conditioned media were measured using ELISA. The level of sAPPα was significantly increased by E144 (n = 4). The level of sAPPβ was decreased by E144, but without significace (n = 4).

Supplementary Fig. 2

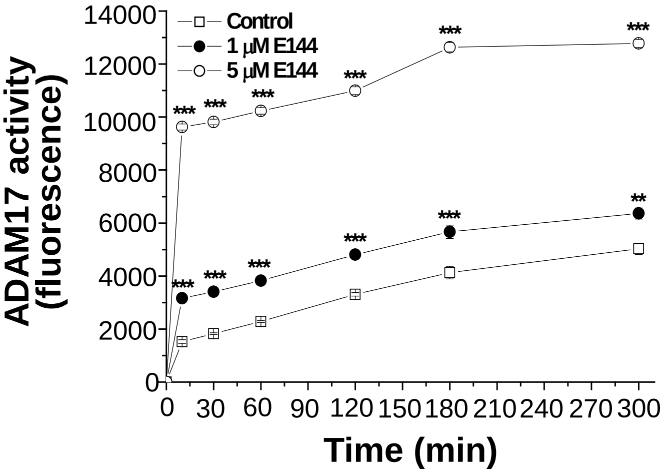

Supplementary Figure 2. E144 increases ADAM17 activity in a time-dependent manner. Human recombinant ADAM17 was used to measure ADAM17 activity as described in Materials and Methods. The presence of 1 or 5 μM E144 significantly increased ADAM17 activity in cell-free assay (n = 4). **, P<0.01; ***, P<0.001.

Supplementary Fig. 3

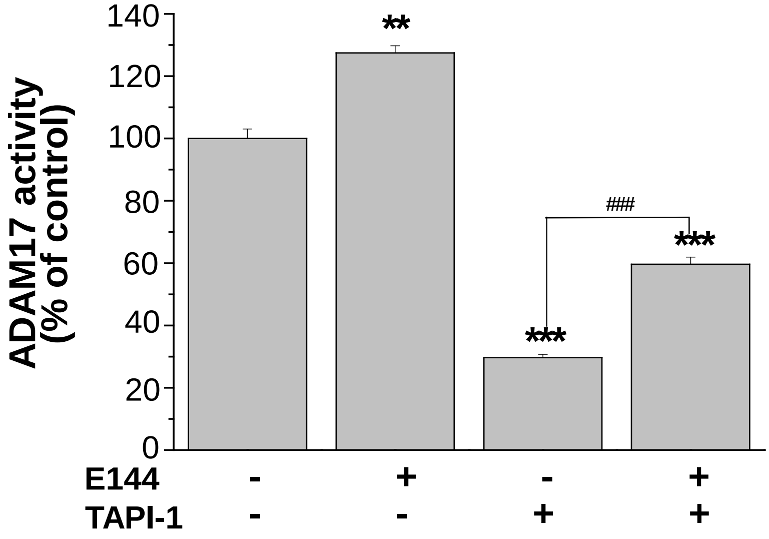

Supplementary Figure 3. The effect of E144 on ADAM17 activation is not abolished by a ADAM17 inhibitor, TAPI-1. Human recombinant ADAM17 was used to measure ADAM17 activity. The presence of 1 μM E144 increased ADAM17 activity while 50 μM TAPI-1 inhibited ADAM17 activity. However, the presence of TAPI-1 did not prevent the effect of E144 on ADAM17 activity (n = 4). **, P<0.01 ***, P<0.001 compared to control. ###, P<0.001 compared to TAPI-1-treated group.

Supplementary Fig. 4

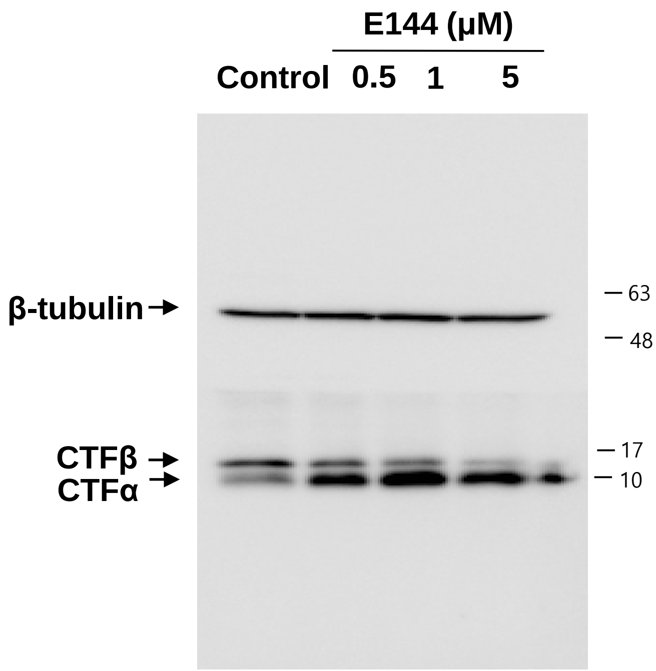

Supplementary Figure 4. Full image of Western blotting for Fig. 2c.

Supplementary Fig. 5

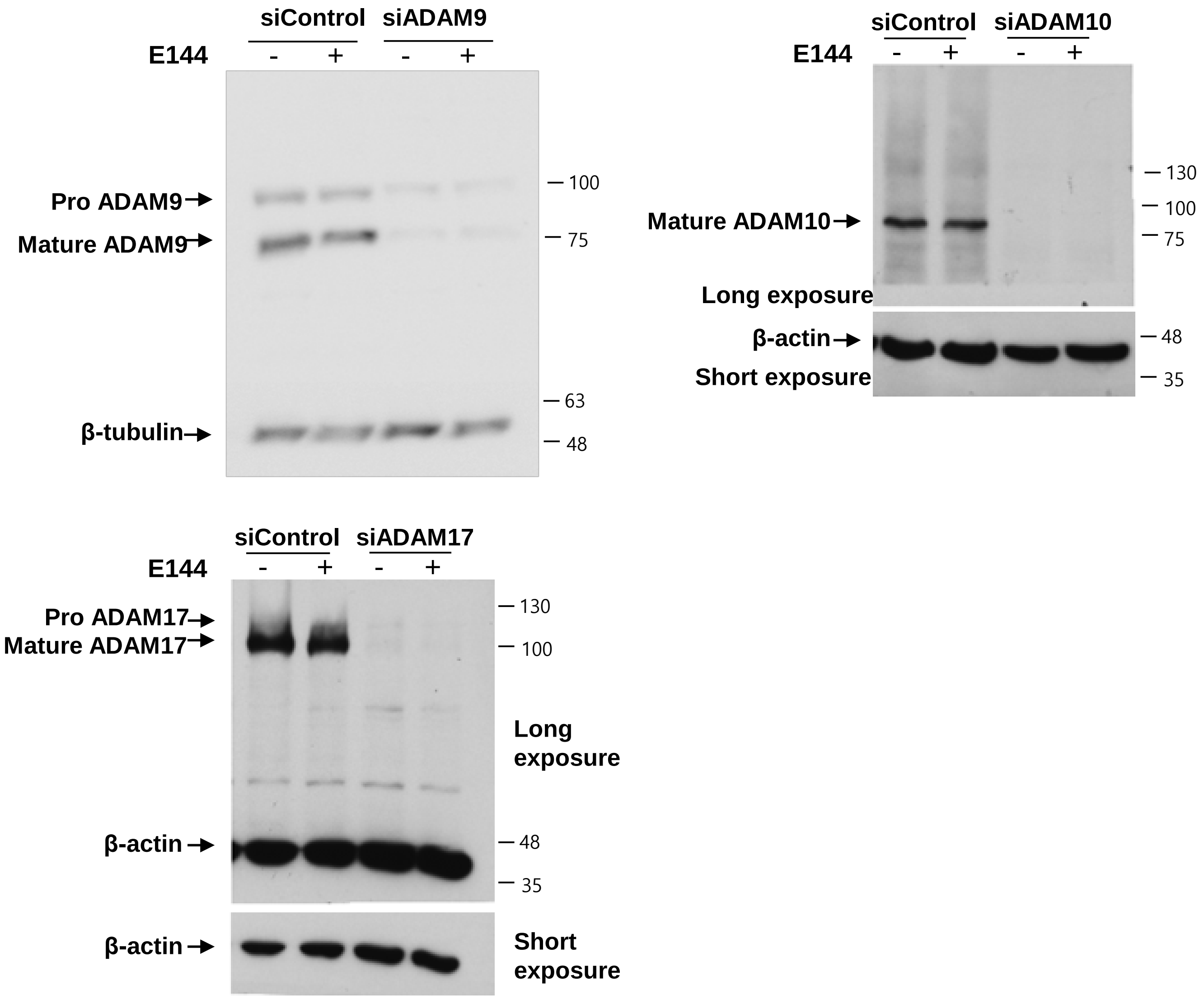

Supplementary Figure 5. Full image of Western blotting for Fig. 6a.

Supplementary Fig. 6

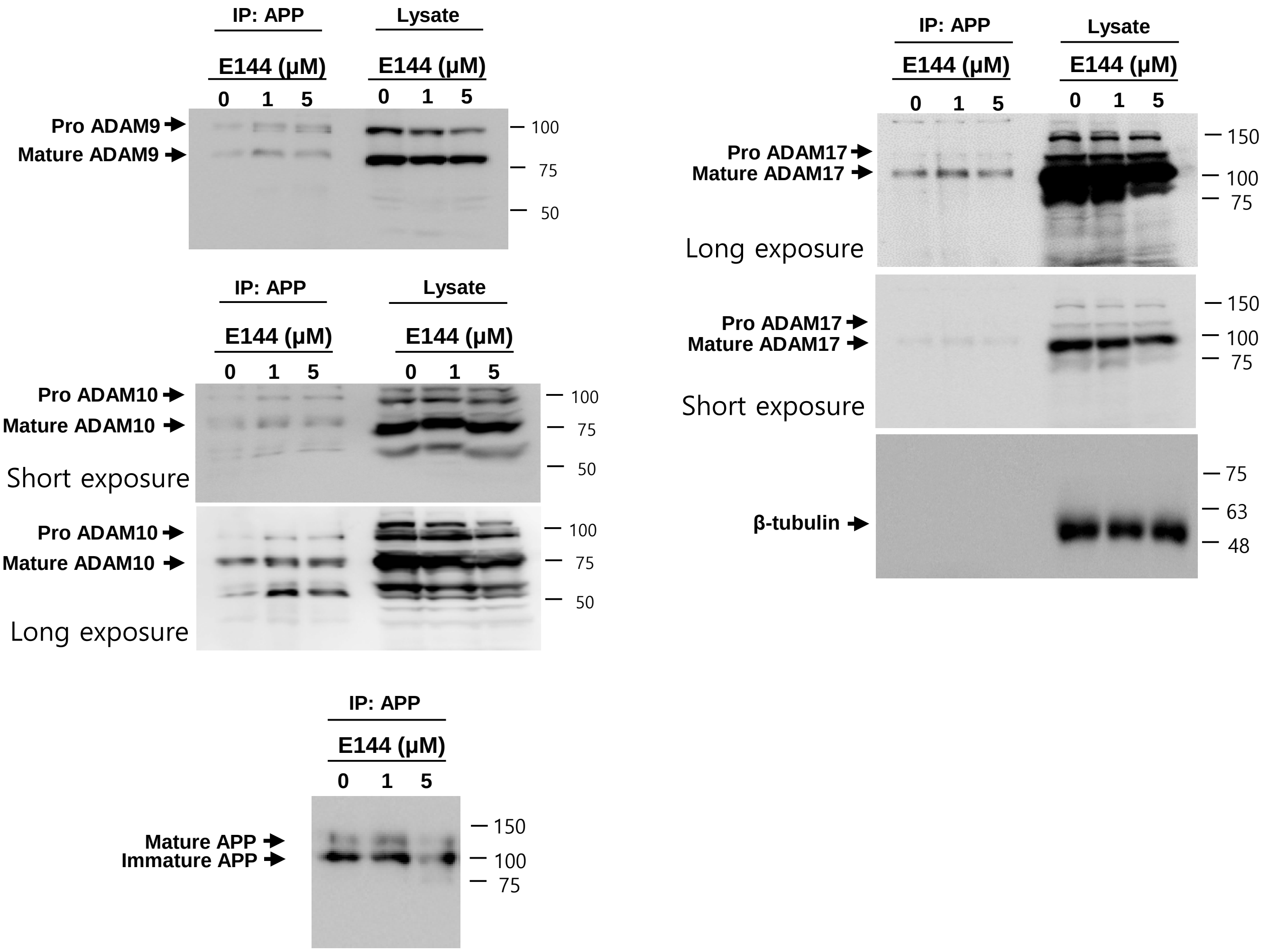

Supplementary Figure 6. Full image of Western blotting for Fig. 7a.
